# Complex life-cycles in trophically transmitted helminths: Do the benefits of increased growth and transmission outweigh generalism and complexity costs?

**DOI:** 10.1016/j.crpvbd.2022.100085

**Published:** 2022-03-01

**Authors:** Daniel P. Benesh, James C. Chubb, Kevin D. Lafferty, Geoff A. Parker

**Affiliations:** a Humboldt University of Berlin, Molecular Parasitology, Philippstr. 13, Haus 14, 10115, Berlin, Germany; b Leibniz-Institute of Freshwater Ecology and Inland Fisheries (IGB), Müggelseedamm 310, 12587, Berlin, Germany; c Department of Evolution, Ecology and Behaviour, University of Liverpool, Liverpool, L69 7ZB, UK; d Western Ecological Research Center, U.S. Geological Survey, at Marine Science Institute, University of California, Santa Barbara, CA, 93106, USA

**Keywords:** Adaptation, Comparative analysis, Complex life-cycle, Host specificity, Life-history strategy, Trophic transmission

## Abstract

Why do so many parasitic worms have complex life-cycles? A complex life-cycle has at least two hypothesized costs: (i) worms with longer life-cycles, i.e. more successive hosts, must be generalists at the species level, which might reduce lifetime survival or growth, and (ii) each required host transition adds to the risk that a worm will fail to complete its life-cycle. Comparing hundreds of trophically transmitted acanthocephalan, cestode, and nematode species with different life-cycles suggests these costs are weaker than expected. Helminths with longer cycles exhibit higher species-level generalism without impaired lifetime growth. Further, risk in complex life-cycles is mitigated by increasing establishment rates in each successive host. Two benefits of longer cycles are transmission and production. Longer cycles normally include smaller (and thus more abundant) first hosts that are likely to consume parasite propagules, as well as bigger (and longer-lived) definitive hosts, in which adult worms grow to larger and presumably more fecund reproductive sizes. Additional factors, like host immunity or dispersal, may also play a role, but are harder to address. Given the ubiquity of complex life-cycles, the benefits of incorporating or retaining hosts in a cycle must often exceed the costs.

## Highlights

• Comparing helminth species hints at the costs and benefits of complex life-cycles.
• Generalism and survival costs in longer life-cycles are weaker than expected.
• Longer life-cycles have growth and transmission benefits.
• The benefits of a complex life-cycle must often exceed the costs.

## Graphical abstract

**Figure:**
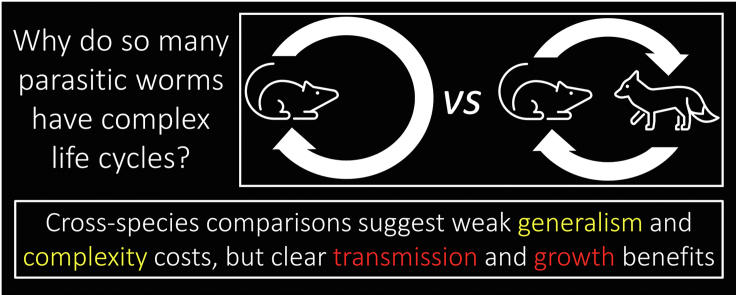

## 1 Introduction

Parasitic worms (helminths) typically have complex life-cycles (CLCs) in which they infect multiple hosts in succession before reproducing. Seal worms (*Pseudoterranova* spp.) are an extreme example - they infect up to five different hosts before reproducing! At first glance, CLCs seem risky. Consider a simple formulation for life-time reproductive success F in a two-host CLC:

(1) $${\text{F}}_{\text{complex, ij}}={\text{P}}_{\text{i}2}{\text{P}}_{\text{j}2}{\text{L}}_{\text{j}}{\text{bW}}_{\text{j}}\text{/}\left(1+\text{c}\right)$$

where P_i2_ is the probability of a parasite propagule (egg or larva free in the environment) encountering, infecting, and developing to infectivity in the intermediate (prey) host i, P_j2_ is the probability of an infective larva in i encountering, infecting, and developing to sexual maturity in the definitive (predator) host j, W_j_ and L_j_ are reproductive size and longevity in host j, and b is a constant relating the rate of egg production to body size W (body size is proportional to fecundity in helminths). Since CLCs involve multiple hosts, we also include a generalism cost c. For a direct cycle in either of the two hosts, F is:

(2a) $${\text{F}}_{\text{simple, i}}={\text{P}}_{\text{i}1}{\text{L}}_{\text{i}}{\text{bW}}_{\text{i}}$$

(2b) $${\text{F}}_{\text{simple, j}}={\text{P}}_{\text{j}1}{\text{L}}_{\text{j}}{\text{bW}}_{\text{j}}$$

where P_i1_ and P_j1_ are the probabilities of completing the entire one-host cycle in prey host i or predator host j.

These equations do not cover predicted transitional states between simple and CLCs (e.g. reproducing in both hosts; Choisy et al., 2003; Parker et al., 2003; Iwasa & Wada, 2006; Ball et al., 2008), so they should not be considered as conditions for *evolving* CLCs. Rather, they are intended to illustrate the pros and cons of *having* CLCs.

Two potential disadvantages of CLCs are evident. First, infecting hosts with different physiologies, immune systems, and often body temperatures may entail generalism costs (c > 0) that reduce survival and/or growth. Second, there are more opportunities to die before reproducing in a CLC, as parasites must survive two transmission steps (P_i2_P_j2_) instead of one (P_i1_ or P_j1_). Both costs should rise with the number of successive hosts in a life-cycle.

Despite this, CLCs are ubiquitous in multiple helminth taxa. Helminths are usually trophically transmitted, and prey intermediate hosts may consume more parasite propagules than predator definitive hosts (P_i2_ > P_j1_), increasing net transmission and survival in CLCs (P_i2_P_j2_). This is thought to favor adding or retaining intermediate hosts in life-cycles, i.e. downward host incorporation (Parker et al., 2003). Upward incorporation, by contrast, adds higher-level predators as definitive hosts. Potential benefits to adding a bigger, longer-lived predator are avoiding mortality from predation (P_i2_ > P_i1_), living longer as adults (L_j_ > L_i_), and growing to larger, more fecund reproductive sizes (W_j_ > W_i_). Thus, two major drivers for evolving and maintaining CLCs are increased production (upward incorporation) and increased transmission (downward incorporation), either of which may increase in longer CLCs with more successive hosts.

Life-cycles of nearly 1000 trophically transmitted acanthocephalans, cestodes, and nematodes have been collated into a comprehensive database (Benesh et al., 2017). We review new analyses with this dataset comparing species with different life-cycles. Such comparisons cannot determine whether CLCs originated *via* upward or downward incorporation, but they can help us understand why the benefits appear to exceed the costs in parasites that have evolved CLCs.

## 2 Cost of CLC: Generalism

**Fig. 1:**
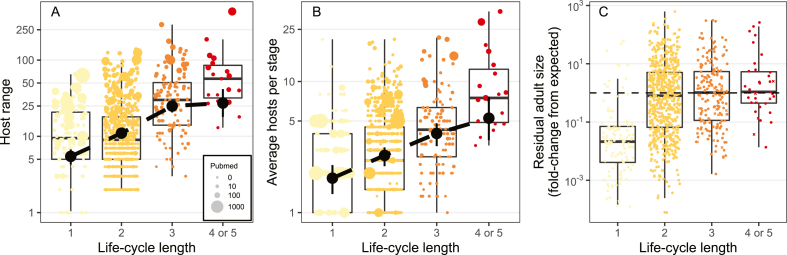
Species-level host generalism (number of known hosts) (**A**), average generalism per stage (**B**), and residual reproductive size after adjusting for host mass (**C**) as a function of life-cycle length. In **A** and **B**, colored points are species values scaled by study effort. Black points are estimated means and 95% credible intervals from models controlling for study effort and taxonomy. In **C**, circles and Xʼs are observed and imputed data, respectively. **A** and **B** adapted from Benesh et al. (2021b) and **C** from Benesh et al. (2021a).

A cost of generalism could be an organism-level constraint on the total host range leading to trade-offs between stages (Gandon, 2004), such as if high generalism at one stage (e.g. intermediate host) limits generalism at other stages (e.g. definitive host) (Palm & Caira, 2008), thereby reducing transmission opportunities (lower P_i2_P_j2_). This scenario predicts a decline in average generalism per stage with life-cycle length and negative correlations between stages. Benesh et al. (2021b) quantified generalism, controlling for study effort, for 842 species using over 17,000 host-parasite records, finding helminths with longer CLCs have higher generalism overall (Fig. 1A). Average generalism per stage increases with life-cycle length (Fig. 1B), and there are not trade-offs in generalism from one stage to the next, which does not support an organism-level limit to generalism. Furthermore, parasites with longer CLCs grow to reproductive sizes as large as expected from definitive host size (Fig. 1C), suggesting that for CLC parasites that infect dissimilar hosts, the resultant high species-level generalism does not impair lifetime growth (c ∼ 0).

Rather than a species-level constraint, generalism might evolve independently in each parasite stage. Generalism is more beneficial in stages with many potential host species; it is highest for second and third intermediate hosts of three- and four-host life-cycles, which are often paratenic hosts (Benesh et al., 2021b). By simulating life-cycles in real food webs, Benesh et al. (2021b) confirmed that these “middle” stages have more potential host species to infect, suggesting that ecological opportunity determines generalism. However, parasites usually infect fewer host species than expected from simulated cycles, hinting at costs. Consistent with a trade-off, helminths spend less time growing and developing in stages where they infect more taxonomically diverse hosts. Acquiring nutrients and avoiding immunity during prolonged growth seems to require specialization, whereas encysting with little growth may be feasible in numerous host species. Sacrificing growth for generalism in some stages may increase transmission opportunities (P_i2_P_j2_) without impairing lifetime growth (W_j_; Fig. 1C), suggesting that generalism costs at the stage-level need not cause an overall performance cost in CLCs (c ∼ 0; of course, parasites failing to evolve CLCs might be those where this cost is high).

## 3 Cost of CLC: Transmission risk

**Fig. 2:**
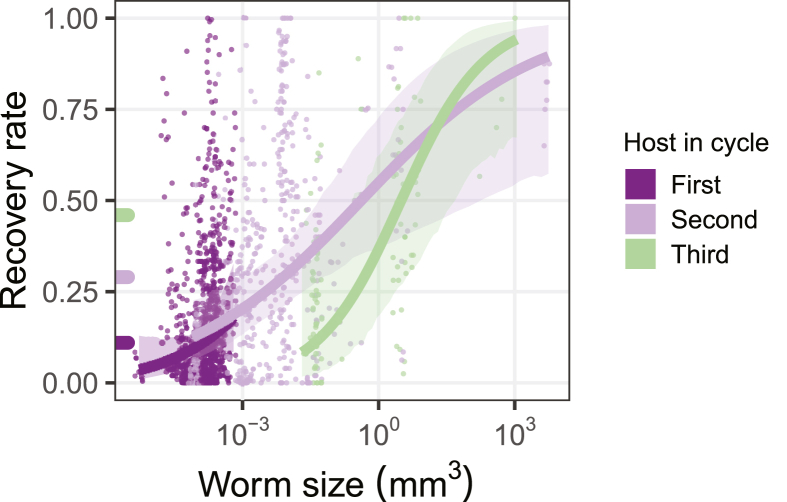
Recovery rate as a function of helminth size. Colors indicate whether the first, second or third host in the life-cycle was exposed (number of parasite species, *n* = 54, 60 and 14, respectively). Lines and 95% credible intervals were estimated with models accounting for variation among studies. Colored ticks along the *y*-axis are model-estimated group averages. Adapted from Froelick et al. (2021).

At each step in a CLC, worms may fail to transmit to the next host, but this risk can be partially mitigated with high establishment rates. Recovery rates from experimental infections (i.e. the proportion of parasites recovered from an administered dose) increase with life-cycle progression: an average helminth had an 11%, 29%, and 46% chance of establishing in the first, second, and third hosts, respectively (Froelick et al., 2021). This trend seems driven by parasite growth: larger larvae from later hosts are more likely to establish infection (Fig. 2). Success in infecting the next host thus increases by growing in intermediate hosts. Such growth may be indispensable given that bigger hosts encountered later in CLCs tend to be less susceptible (Poulin, 2010; Froelick et al., 2021). Parasites can also have dramatically higher infection rates in an upstream host when transmitted *via* a facultative intermediate host *versus* without it (Benesh, 2016). Thus, when transmission to and from an intermediate host is highly efficient, CLCs can have higher overall survival (P_i2_P_j2_ > P_j1_ or P_i1_).

## 4 Benefit of CLC: Propagule transmission

**Fig. 3:**
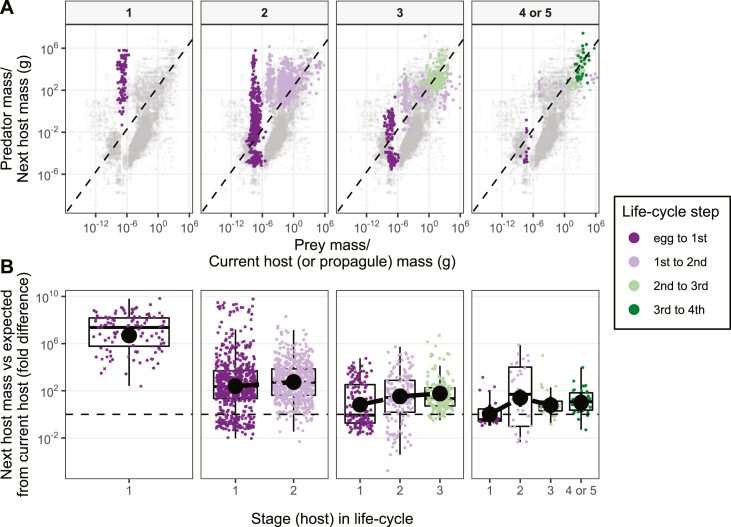
**A** Predator *vs* prey mass. Gray points are a dataset of predator and prey masses (Brose et al., 2019). The dashed line (major axis regression) represents typical predator-prey mass ratios. Trophic links exploited by parasites are overlaid as colored points with current host (or propagule) mass as prey and next host mass as predator. Panels separate parasites by life-cycle length (i.e. the number of successive hosts before reproduction). **B** The difference between observed and expected next host mass (i.e. the residuals from **A**) plotted by life-cycle stage. Black point ranges are means and 95% credible intervals estimated from models accounting for parasite taxonomy and missing data. Missing host masses were imputed; Xʼs represent the average from 100 imputations of the *y*-axis variable. Circles are observed data. Adapted from Benesh et al. (2021a).

Although parasites with CLCs must survive multiple transmission steps, some hosts may be more easily reached in two steps than one (P_i2_P_j2_ > P_j1_), such as if intermediate hosts are more likely to consume parasite propagules (P_i2_ > P_j1_). Benesh et al. (2021a) compared first-host-to-propagule mass ratios with typical predator-prey mass ratios. Parasites with longer CLCs have smaller first hosts (Fig. 3); the first hosts in three- and four-host life-cycles are > 100,000 times smaller than the first (and only) host in one-host cycles, and they are probably > 100,000 times as abundant (Hatton et al., 2019). Propagules are normal-sized food for the small first hosts in longer CLCs, whereas the large first hosts in one-host cycles probably consume propagules accidentally (Fig. 3), especially if they avoid foraging near egg-laden faeces (Chubb et al., 2020). By first infecting smaller, more abundant hosts, longer CLCs become bridges to hard-to-reach hosts.

## 5 Benefit of CLC: Reproductive size

**Fig. 4:**
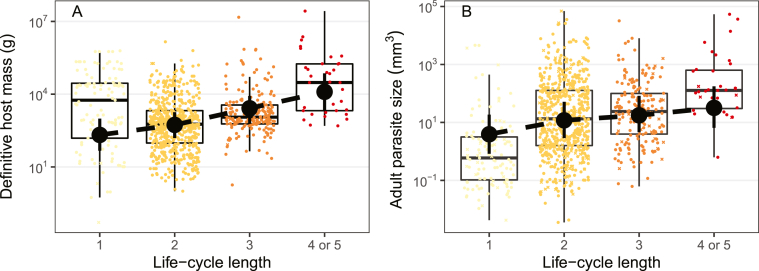
Definitive host mass (**A**) and reproductive size (**B**) for helminths with different life-cycle lengths. Point ranges are means and 95% credible intervals estimated from models accounting for parasite taxonomy and missing data. In all panels, Xʼs represent imputed data; circles represent observed data. Adapted from Benesh et al. (2021a).

Although small hosts aid transmission, they constrain growth. Helminths typically grow larger in big hosts, particularly endotherms (Benesh et al., 2021a and references therein), where they have more space, time, and energy to grow. In longer CLCs, parasites infect larger final hosts (Fig. 4A); mean definitive host mass in four-host cycles was estimated as ∼60-fold that in one-host cycles, implying a ∼20-fold to 60-fold higher host metabolic rate and a ∼2-fold to 3-fold longer host lifespan (Hatton et al., 2019). Accordingly, helminths with longer CLCs grow to larger sizes at reproduction (W_j_ > W_i_; Fig. 4B).

## 6 Conclusions and future directions

**Fig. 5:**
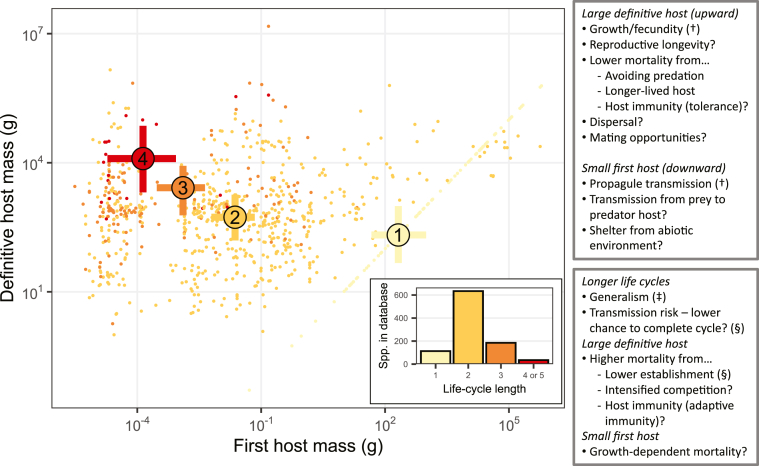
Definitive *vs* first host mass for helminths in the life-cycle database (Benesh et al., 2017). Small dots are species. Large dots are estimated means and 95% credible intervals for helminths with one-, two-, three-, and four-host cycles (see Fig. 3, Fig. 4). The inset shows the frequency distribution of life-cycle lengths in the database. The upper right box lists potential benefits of adding/maintaining larger predators as definitive hosts or smaller prey as intermediate hosts (i.e. of having a longer life-cycle). The lower box lists potential costs to infecting such hosts as well as costs associated with longer cycles generally. Benefits and costs highlighted in this review are noted: † Benesh et al., (2021a); ‡ Benesh et al. (2021b); § Froelick et al. (2021).

The best hosts for growth and reproduction (big endotherms) are not the best for propagule transmission (small, abundant hosts) (Benesh et al., 2021a). Bridging the gap from particularly small first hosts to especially large definitive hosts may require several intermediate hosts and a longer CLC. This inability to maximize transmission and growth with a single host (P_i2_ > P_j1_ but W_i_ < W_j_) likely explains the ubiquity of CLCs in helminths (Fig. 5). Furthermore, two presumed costs of CLCs may not be as bad as assumed. First, risk from multiple transmission steps (P_i2_P_j2_) is partly mitigated by increasing establishment rates. Second, although parasites with longer CLCs are generalists at the species level, this does not impair their overall growth (c ∼ 0). Rather, these parasites seem to sacrifice growth for higher generalism in the life stages that encounter diverse hosts (Benesh et al., 2021b). Such results are consistent with the idea that CLCs are common because they are adaptive for many parasites.

Questions remain that cannot be answered with the database (Fig. 5), especially concerning parasite mortality and the probability of completing longer *vs* shorter life-cycles (P_i2_P_j2_ *vs* P_i1_ or P_j1_). Upward incorporation of large predators may decrease parasite mortality, both because parasites avoid predation and because the final host is less likely to die, but, although larger hosts live longer, it is not certain that parasites live longer in them (L_j_ > L_i_; the database contains prepatent periods but not longevity). Larger hosts are less susceptible to infection (Froelick et al., 2021) and competition intensifies in large, top predators, as they commonly harbor bigger (Poulin & George-Nascimento, 2007), more aggregated (Lester & Mcvinish, 2016), and more diverse parasite communities (Arneberg, 2002). Further, immune-dependent mortality might increase in larger hosts with more sophisticated immune systems (e.g. acquired immunity in vertebrates), but may also decline if long-lived hosts tolerate infections and avoid pathological immune responses (Brace et al., 2017). Downward incorporation of small prey hosts, besides decreasing propagule mortality, may also shield parasites from abiotic conditions, e.g. through host mechanisms to maintain homeostasis, like thermoregulation (Molnár et al., 2013). Then again, small hosts have short life expectancies that could be further shortened by growing worms (Ball et al., 2008). Transmission rates between successive hosts likely vary with life-cycle length. Longer CLCs were associated with smaller predator-prey mass ratios (Fig. 3), which could either increase transmission (small predators are relatively more abundant) or reduce transmission (small predators consuming relatively large prey can meet their energy demands with lower feeding rates). Comparing parasite mortality and transmission among hosts within cycles (P_i2_ *vs* P_j2_), as well as between different kinds of cycles (P_j1_ *vs* P_i2_P_j2_), will provide further insight into CLC evolution.

Besides higher transmission or fecundity, CLCs may have other benefits. For example, some parasites undergo little growth in large definitive hosts (Benesh et al., 2013), so why are such hosts retained in the life-cycle? One possibility is that, since large predators consume multiple infected prey items, parasites with CLCs may encounter more potential mates and avoid inbreeding (Brown et al., 2001; Rauch et al., 2005). Parasites with longer CLCs and larger definitive hosts may also have higher dispersal. Dispersal may be particularly advantageous if hosts at some life stages are prone to local extinctions (Rudolf & Lafferty, 2011) or if a mobile, predator host is attracted to areas with a high abundance of suitable prey intermediate hosts. Yet, mobile hosts could also deposit parasite propagules in unsuitable habitats. How dispersal and mating help maintain large, mobile hosts in CLCs deserves exploration, particularly as they affect parasites’ ability to survive in and adapt to changing environments.

Finally, life-cycle knowledge remains biased. The database likely overestimates and underestimates the frequency of one-host and four-host cycles, respectively, because it contains more mammal helminths (most one-host cycles involve mammals) and fewer marine helminths (which traverse longer food chains) than expected (Froelick et al., 2021). Filling such knowledge gaps should remain a goal (Blasco-Costa & Poulin, 2017), as well as confirming trends in taxa not included in the database, like trematodes. Only by documenting this diversity can we understand which life-cycles evolve and persist and which do not.

## Funding

Daniel P. Benesh was funded by 10.13039/501100001659Deutsche Forschungsgemeinschaft project BE 5336/3-1. Additional support was provided by the U.S. Geological Survey Ecosystems Mission Area.

## CRediT author statement

Daniel P. Benesh: conceptualization, formal analysis, visualization, funding acquisition, writing - original draft, writing - review & editing. James C. Chubb: conceptualization, writing - review & editing. Kevin D. Lafferty: conceptualization, writing - review & editing. Geoff A. Parker: conceptualization, writing - review & editing. All authors read and approved the final manuscript.

## Declaration of competing interests

The authors declare that they have no known competing financial interests or personal relationships that could have appeared to influence the work reported in this paper.
